# Molecular characterization and integrative genomic analysis of a panel of newly established penile cancer cell lines

**DOI:** 10.1038/s41419-018-0736-1

**Published:** 2018-06-07

**Authors:** Qiang-hua Zhou, Chuang-zhong Deng, Zai-shang Li, Jie-ping Chen, Kai Yao, Kang-bo Huang, Ting-yu Liu, Zhuo-wei Liu, Zi-ke Qin, Fang-jian Zhou, Wenlin Huang, Hui Han, Ran-yi Liu

**Affiliations:** 1 Sun Yat-Sen University Cancer Center, State Key Laboratory of Oncology in South China, Collaborative Innovation Center of Cancer Medicine, 510060 Guangzhou, China; 2 Department of Urology, Sun Yat-Sen University Cancer Center, 510060 Guangzhou, China; 3 Department of Urology, Shenzhen People’s Hospital, Jinan University, 518020 Shenzhen, China; 4 Guangdong Provincial Key Laboratory of Tumor Targeted Drugs and Guangzhou Enterprise Key Laboratory of Gene Medicine, Guangzhou Doublle Bioproducts Co. Ltd., 510663 Guangzhou, China

## Abstract

Cell line models are essential tools to study the molecular mechanisms underlying tumor initiation and progression. There are limited treatment options for penile squamous cell carcinoma (PSCC), accounting for 1–2% of male tumors in developing countries, and limited progress in preclinical research in PSCC due to lacking available models with identified genomic characteristics. Here, biological and molecular characteristics and whole-genomic alterations were analyzed in a panel of PSCC cell lines newly established in our laboratory. These cell lines were all human papillomavirus (HPV)-negative, epithelial-like, immortalized, and tumorigenic in nude mice, whereas they displayed different proliferation, migration and invasion capacities in vitro, and tumorigenic ability in nude mice. They were all cisplatin sensitive, anti-EGFR therapy resistant, and androgen irresponsive. Whole-genomic sequecing analysis revealed that transition mutations (C:G>T:A and T:A>C:G) were the most common substitution types in these cell lines, whereas *ERCC5*, *TP53*, *PTH1*, *CLTCL1*, *NOTCH2*, *MAP2K3*, *CDK11A/B*, *USP6*, *ADCH5*, *BCLAF1*, *CDKN2A*, *FANCD2*, *HRAS*, and *NOTCH1* were the most frequently altered genes. Amplifications of *MYC*, *PLAG1*, *NCOA2*, *RUNX1T1*, *COX6C*, and *EGFR* and losses of *FBXW7*, *TET2*, *XPC*, and *FANCE* were frequently observed in cell lines. The exomic variations between cell lines and their corresponding cancer tissues were highly consistent. Genetic variations were mainly involved in the MAPK, Jak-STAT, TGF-beta, Notch, and apoptosis signaling pathways. Conclusively, these panel of PSCC cell lines established in our laboratory harbor some common or specific biological characteristics and genomic variations, and they may serve as optimal models to investigate the molecular mechanisms underlying the progression, metastasis, relapses, and treatment resistance of PSCC and to develop effective treatment strategy.

## Introduction

Penile squamous cell carcinoma (PSCC) is a rare malignancy in developed countries with an incidence of (0.3–1)/100,000. However, PSCC constitutes up to 1–2% of male cancer in the developing world^1–3^. Risk factors for PSCC include poor hygiene, phimosis, smoking, lack of circumcision, and human papillomavirus (HPV) infection^1^. The 5-year overall survival in patients with metastatic inguinal lymph nodes (LNs) is approximately 50% and with pelvic LN metastasis is ~0%, compared with >90% in patients without LN involvement^4–6^. Due to the limited basic and clinical studies, treatment options are very deficient for PSCC patients, especially those with advanced disease.

Previous studies have revealed the mutational landscapes of PSCC using next-generation sequencing, which contributes greatly to our understanding of the tumorigenesis and development of penile cancer^7–9^. However, the biological functions of these genetic aberrations have not been clarified by in vitro and animal experiments, due to fewer available PSCC cell lines with various genetic backgrounds. Japanese researchers established three penile cancer cell lines in the last century^10–12^, and then a pair of cell lines Ki-PeCa-P1/L1 from a primary lesion and its corresponding metastatic LN^13^ and a cell line from human verrucous penile carcinoma^14^ were established by German and Brazilian researchers, respectively, in 2012 and 2016. Even so, few findings about molecular mechanisms underlying PeSCC oncogenesis based on these cell lines were reported in subsequent years.

To investigate molecular mechanisms underlying PSCC oncogenesis, we established four other new PSCC cell lines after development of an HPV-negative PSCC cell line with a TP53 mutation in 2016^15^, and compared the biological and genetic characteristics of these five cell lines; some common or specific genomic variations were found, which will contribute to clarifying molecular mechanisms of PSCC tumorigenesis and searching for potential therapeutic targets of PSCC in further studies.

## Results

### The origins of cell lines

All cell lines were derived from Chinese PSCC patients, whose detailed clinicopathological characteristics are presented in Additional Table S1. Notably, 149RCa and 149RM were two different cell lines generated from the local recurrent lesion or a nearby scrotal invasion lesion in the same patient who suffered a relapse disease after systemic therapy. Cell line LM156 was generated from a patient with poorly differentiated disease.

### Morphological features and purity of cell lines

**Fig. 1:**
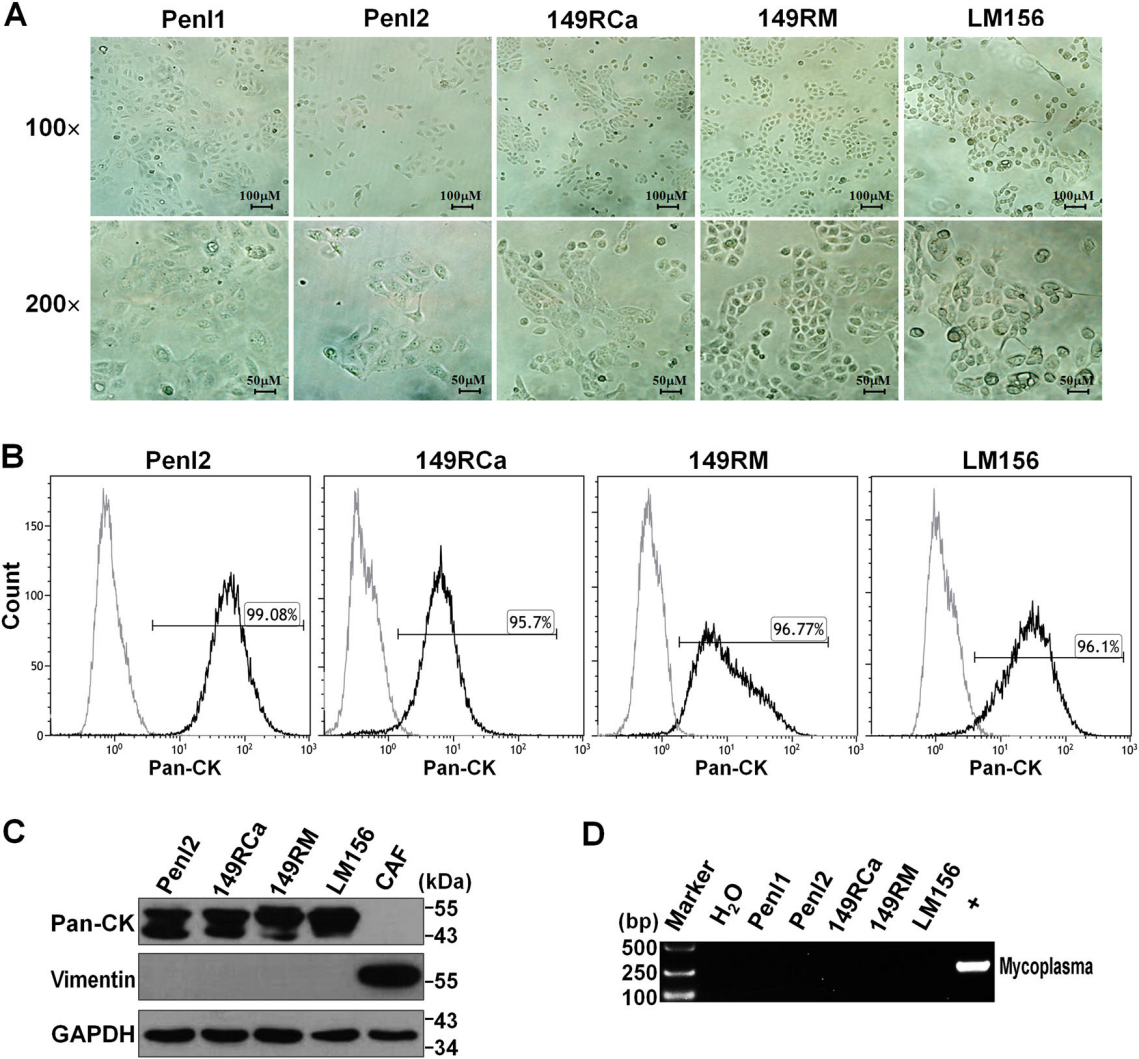
Morphology and purity of the cell lines, and PCR detection for HPV and mycoplasma. **a** Phase-contrast photomicrograph of cell lines. **b** Flow cytometry assay for Pan-CK expression, indicating the purity of new cell lines. **c** Western blotting (WB) assay for the expression of Pan-CK and Vimentin (cancer-associated fibroblast (CAF) was used as a control). **d** PCR detection of mycoplasma contamination in cell lines (+, positive control)

In vitro culture showed that all PSCC cell lines grew well as a well-attached monolayer, displayed a distinct epithelial appearance (with a cobblestone pattern), and exhibited typical characteristics of malignant change: large nucleus, scarce cytoplasm, prominent nucleoli, irregular size and shape (Fig. 1a). Flow cytometry assays revealed that 95.7–99.08% of the cells were positive for epithelial cell marker Pan-cytokeratin (Pan-CK, Fig. 1b), and Western blotting (WB) assays showed that Pan-CK was highly expressed in all PSCC cell lines, whereas fibroblast marker vimentin could not be detected (Fig. 1c). These data suggested that these five cell lines were highly purified, without fibroblast contamination.

### Detection for the infection of HPV and mycoplasma

HPV infection is thought to play an important role in the carcinogenesis of PSCC^16^; however, HPV DNA could not be detected in all cell lines and their corresponding tumor tissues (quantitative real-time PCR, all Ct values > 35) (Additional Table S1). In addition, none of the cell lines were found to contaminate with mycoplasma (Fig. 1d).

### Short tandem repeat (STR) analysis for the validation of cell line

Detailed STR information of the cell lines was presented in Additional Table S2. The STR profiles of all five cell lines did not match any cell lines in the databases from ATCC, DSMZ, or JRCB. Notably, the STR profiles of 149RCa and 149RM were identical, confirming that they were from the same patient.

### Cell proliferation in vitro and tumorigenicity in nude mice

**Fig. 2:**
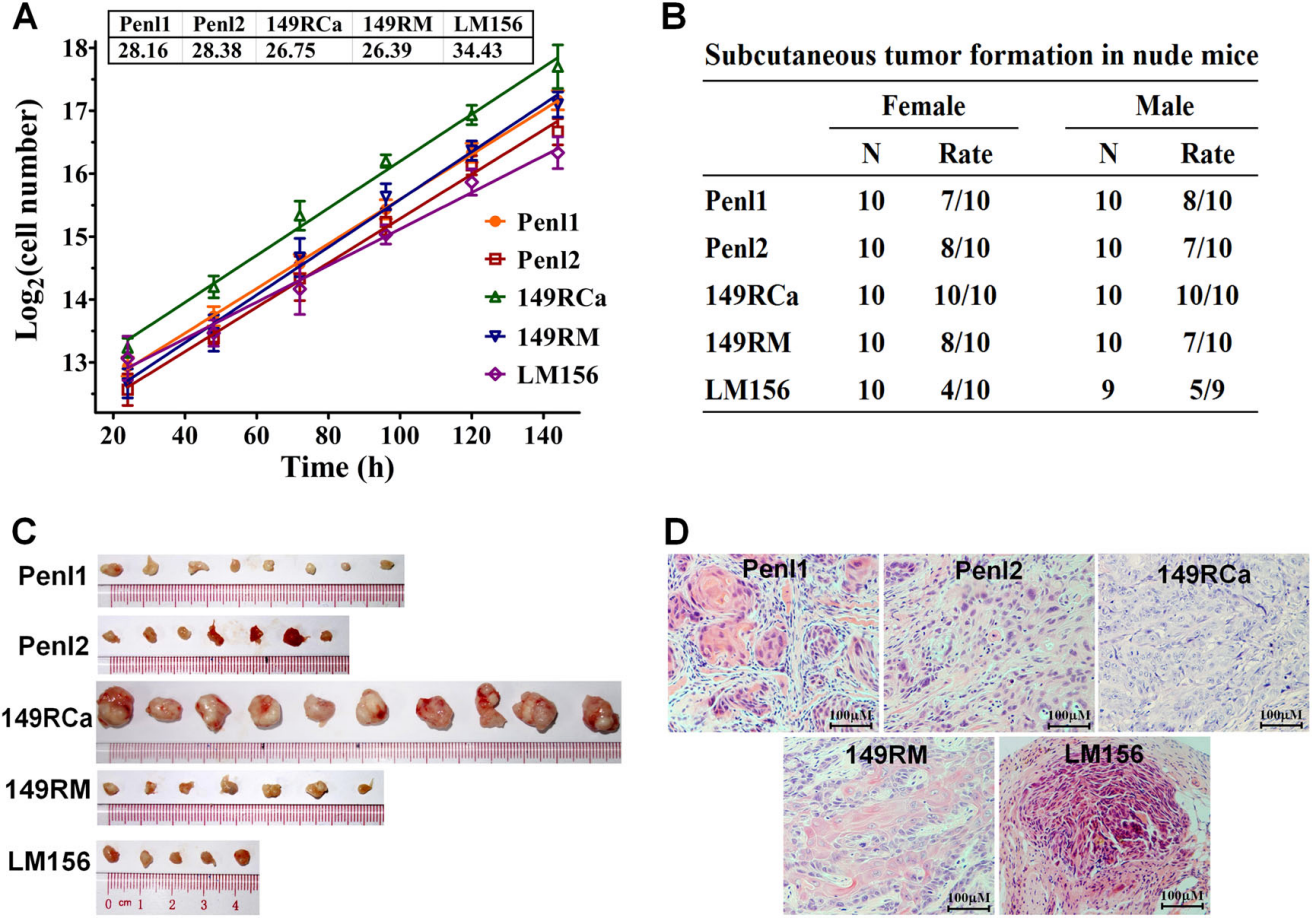
Proliferation in vitro and tumorigenicity in nude mice for PSCC cell lines. **a** Growth curves and corresponding doubling times of PSCC cell lines in Dulbecco’s Modified Eagle Medium supplemented with 10% fetal bovine serum. **b** Tumor formation rates of these cell lines in female/male BALB/c nude mice. **c** Xenograft tumors harvested 3 weeks after tumor cells inoculation (in male nude mice). **d** The corresponding hematoxylin and eosin staining pictures of xenograft tumors, confirming the histology of SCC

All PSCC cell lines were immortalized cells and grew well in Dulbecco’s Modified Eagle Medium (DMEM) supplemented with 10% fetal bovine serum (FBS). The growth curves and doubling times of the cell lines are presented in Fig. 2a. Among the five cell lines, the 149RCa and 149RM cell line had the shortest doubling time (26.75 and 26.39 h), whereas the LM156 cell line had the longest doubling time (34.43 h). These cell lines were all tumorigenic in male/female BALB/C nude mice, but their tumor formation rates were different, ranging from 4/10 to 10/10 after subcutaneous incubation of 5 × 10^6^ cells (Fig. 2b, c and Additional Fig. S1). Cell line 149RCa grew faster than the other four cell lines in nude mice (Fig. 2c and Additional Fig. S1). Hematoxylin and eosin (H&E) staining analysis showed that the xenograft tumors were squamous cell carcinoma, consistent with their original tumors (Fig. 2d and Additional Fig. S1).

### Migration and invasion abilities of PSCC cell lines

**Fig. 3:**
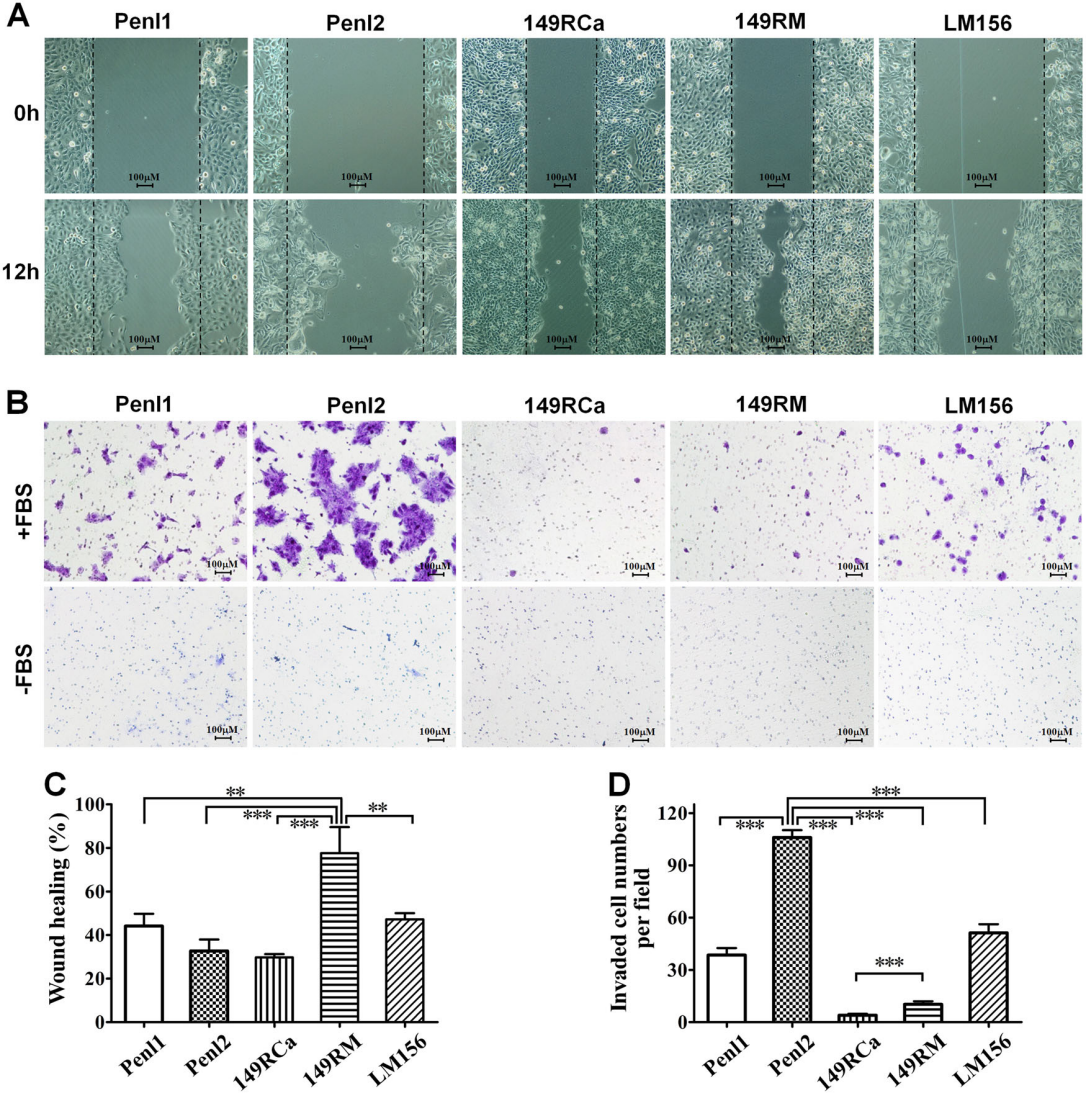
Migration and invasion potential in vitro. **a**, **c** Different migration potentials of the cell lines were detected by a wound healing assay. **a** Representative pictures (100×) of 0 and 12 h after scratching. **c** Statistic results of the percentages of wound healing. **b**, **d** A Transwell assay was conducted to compare the invasive potential of the cell lines. **b** Representative pictures (100×) of the migrated cells (“−FBS”, negative control). **d** Statistic results of the average invasive cells per field. Data shown are mean ± S.D. of three replicates. **p < 0.01, ***p < 0.001

Among these five cell lines, 149RM had the highest migration potential (Fig. 3a, c; *p* < 0.01 or 0.001), whereas Penl1, Penl2, and LM156 that were from metastatic LNs had stronger invasive ability, especially Penl2 (Fig. 3b, d; all *p* < 0.001). Although the invasion rate of 149RM was lower than three cell lines from metastatic LNs, 149RM had stronger invasion activity than 149RCa (Fig. 3d, *p* < 0.001), which may be partially because 149RCa and 149RM are derived from the same patient’s local recurrent lesion and nearby scrotal invasion lesion, respectively.

### Androgen response and treatment sensitivity for therapeutic agents

**Fig. 4:**
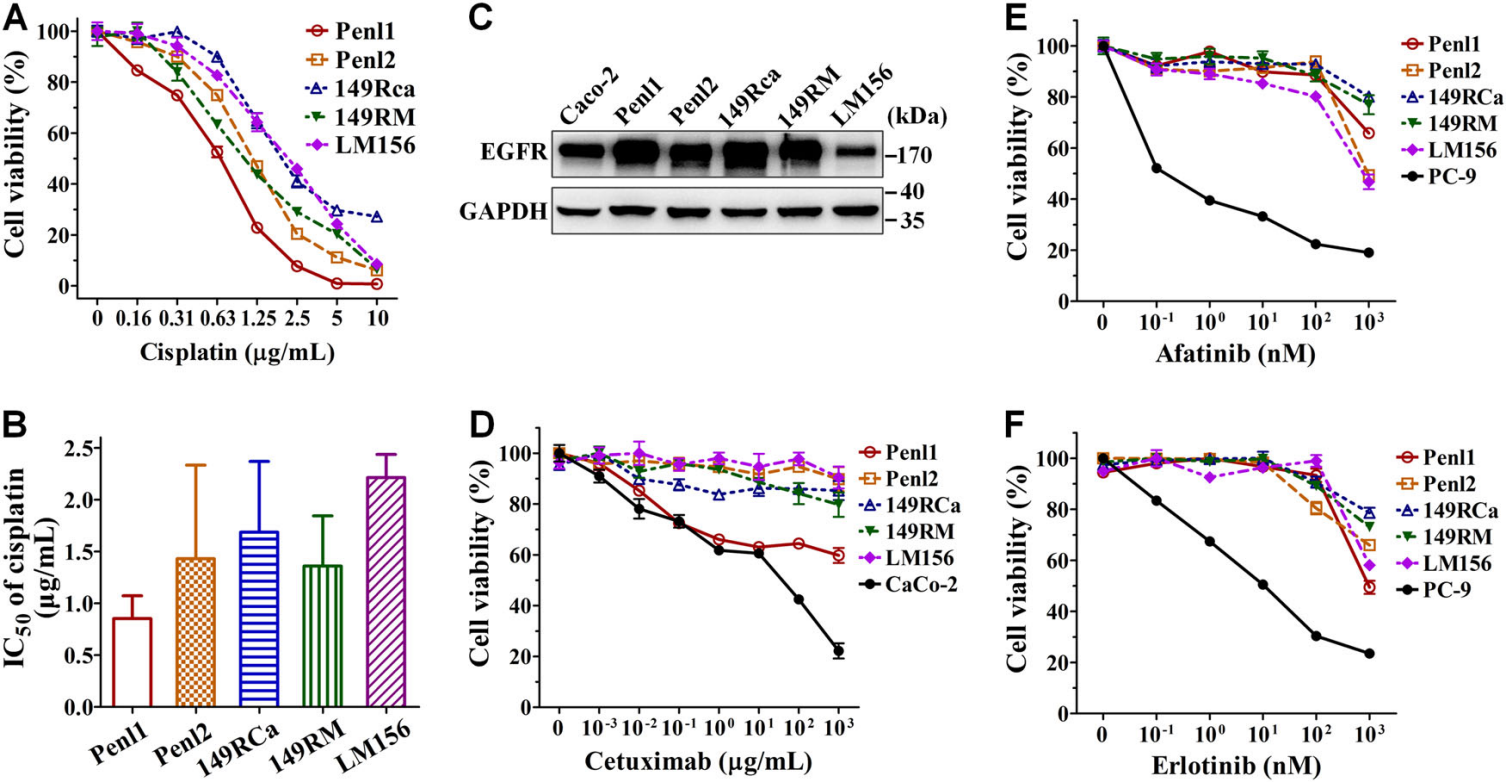
The response of penile cancer cell lines to cisplatin and EGFR-targeted drugs. Cells were treated with cisplatin or EGFR-targeted drugs for 48 h. A CCK-8 assay was used to assess the relative cell viability. **a** Representative cisplatin dose-dependent cell viability. **b** The average IC_50_ values of cisplatin for the PSCC cell lines. **c** WB assays for *EGFR* expression (GAPDH was used as the loading control). **d**–**f** Representative cell viability curve after treatment with Cetuximab (**d**), Afatinib (**e**), or Erlotinib (**f**) (Caco or PC-9 cells were used as positive control)

The progression of some male tumors may be associated with androgen stimulation; however, we found that these PSCC cells expressed very low levels of androgen receptor (AR), and androgens did not affect the growth of these cells (Additional Fig. S2). We next tested their sensitivity to cisplatin, a widely used chemotherapeutic drug. The results demonstrated that all cell lines were highly sensitive to cisplatin, characterized by low values of the half maximal inhibitory concentration (IC_50_) (0.85–2.21 μg/mL) (Fig. 4c, d).

We next examined the expression of epidermal growth factor receptor (*EGFR*) by WB assay and found that *EGFR* highly overexpressed in PSCC cell lines Penl1, Penl2, 149RCa, and 149RM, whereas there was a moderate *EGFR* expression in LM156 cells (Fig. 4c). These findings suggest a promising EGFR-targeted therapy for PSCC. However, cetuximab, an anti-EGFR antibody, showed no obvious inhibitory activities in these cell lines (all IC_50_s > 1000 μg/mL) (Fig. 4d), whereas cetuximab displayed a slight inhibition in Penl1 cells. Similarly, the five cell lines displayed a strong resistance to other two EGFR tyrosine kinase inhibitors, erlotinib and afatinib (Fig. 4e, f).

### Mutation patterns of penile cancer cell lines

**Fig. 5:**
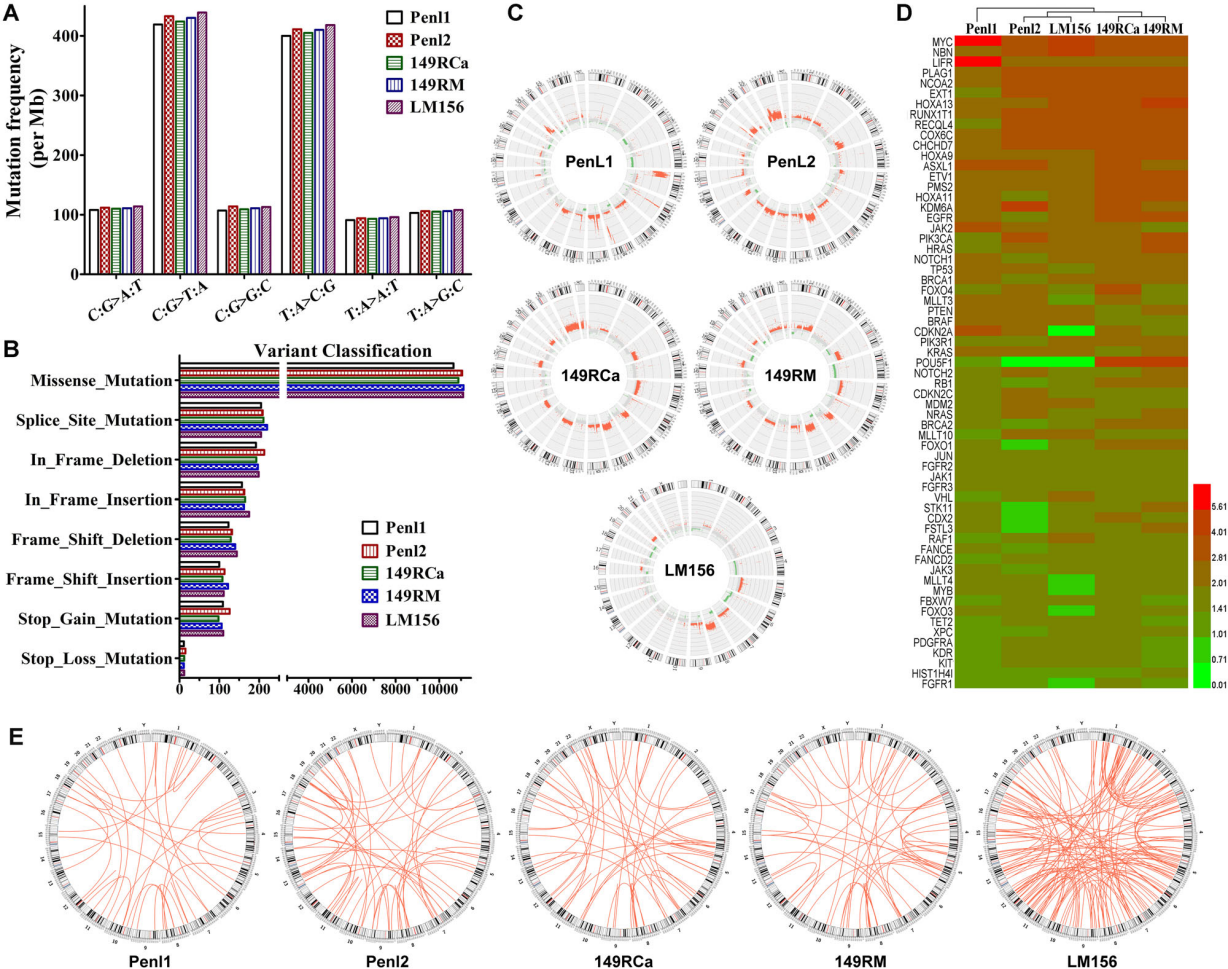
SNVs, InDel, and CNV spectrum in penile cancer cell lines. Whole-genome sequencing (WGS) analysis was performed in five penile cancer cell lines. **a** Mutation frequency for the type of base substitution on the whole-genome scale. **b** Variant classification for protein-altering somatic alterations. **c** Circos plots of CNV profiling (inner circle) for copy number change in the whole genome. Red dots denote copy number gain, and green dots denote copy number loss. **d** Heatmap of copy numbers (CN) for prioritized functional genes. CN ≥2.8 is considered copy number gain (amplification), 2.8>CN>1.4 is considered normal diploid, and CN ≤1.4 is considered copy number loss (deletion). **e** Circos plots showing the SV spectrums in these PSCC cell lines

Whole-genome sequencing (WGS) analyses revealed that these cell lines exhibited a similar mutational spectrum, containing 1229–1290 single-nucleotide polymorphisms (SNPs) per megabyte compared to the National Center for Biotechnology Information (NCBI) human reference genome (hg19), and transition mutations (C:G>T:A and T:A>C:G) were the most frequent substitution types (Fig. 5a and Additional Table S3). The single-nucleotide variations (SNVs) and short insertions/deletions (InDels), resulting in protein variations, included missense, splice site, stop-gain and stop-loss mutations, frame-shift and in-frame InDels; among them, missense mutation was the most common mutation type (Fig. 5b). We compared the exomic SNVs/InDels between cell lines and their corresponding cancer tissues and found that their exomic variations were highly consistent, and the accordance ratios were from 91.4 to 94.7% (Additional Table S4), which suggested that these cell lines were suitable representatives of their corresponding penile cancer tissues.

### Identification of prioritized functional gene mutations in PSCC cell lines

We then analyzed the mutations of common driving or potentially targetable genes with customized filtering criteria as described in Methods. As the data exhibited in Table 1, *TP53*, *PTH1*, *ERCC5*, *CLTCL1*, *USP6*, *NOTCH2*, *ADCH5*, *MAP2K3*, *CDK11A/B*, and *BCLAF1* (5/5), *CDKN2A* and *FANCD2* (4/5), *NOTCH1*, *HRAS*, *KDR*, and *CIITA* (3/5) are the most frequently altered genes, most of which accord with previous reports^7–9^. Among them, 23 were tested by Sanger sequencing, and 22 were validated, except for *NOTCH2*: exon2: c.C238T: p.N46S in cell line 149RM (Table 1, Additional Fig. S3).

**Table 1:** SNVs and InDels of critical genes in penile cancer cell lines

Genes	Penl1	Penl2	149RCa	149RM	LM156
Tumor suppressor					
*TP53*	R244P*, P33R	R234H	R209Q*, R71C*, P33R	R209Q*, R71C*, P33R	P33R
*PTCH1*	T1195S	T1195S	P1315L	P1315L	P1315L
*ERCC5*	G1053R^$^	G1053^$^	R214C^$^, G1053R^$^	R214C^$^, G1053R^$^	G1053R^$^
*CDKN2A*	R80X*	R80X*	H83Y*	H83Y*	–
*FANCD2*	N791S^$^, Splicing	Splicing	Splicing	Splicing	–
*TET2*	–	–	I1762V	I1762V	–
*ATM*	–	R2691C*	–	–	–
*BRCA1*		S1566G			
*FBXW7*	–	–	S25X	–	–
*MSH6*	–	R922Q	–	–	–
Oncogenes					
*CLTCL1*	V1201fs^$^	E458Q^$^, V1201fs^$^	V1201fs^$^	V1201fs^$^	V1201fs^$^
*USP6*	I67M^$^, R68W^$^, R69W^$^	I67M^$^, R68W^$^, R69W^$^, M80I^$^	I67M^$^, R68W^$^, R69W^$^	I67M^$^, R68W^$^, R69W^$^	I67M^$^, R68W^$^, R69W^$^
*NOTCH2*	N46S*	P6fs	N46S, S220R, P227H, T235S	N46S^Δ^	N46S
*NOTCH1*	G427D^$^	C467F^$^	–	–	D1200N*^$^
*HRAS*	–	–	G13V*	G13V*	G13R*
*KDR*			Q472H	Q472H	Q472H
*CIITA*		I729M^$^	Q21H^$^	Q21H^$^	
*NIN*	E1099K^$^		C1036F		
*ERBB2*	Q902X*^$^	–	–	–	R143Q*
*AFF3*	H914Q^$^				H914Q^$^
*NACA*		K416R^$^			K416R^$^
*PCSK7*	V143F^$^				T743N^$^
*AFF3*	H914Q^$^	–	–	–	H914Q^$^
*BCL11A*	–	R249T	–	E539K	–
*ROS1*	–	P224S*^$^	–	–	–
*ALK*	R291H*	–	–	–	–
*ABL1*	–	–	–	–	K404T*^$^
*JAK3*	–	–	–	–	I688F^$^
Protein kinases					
*ADCK5*	L419fs	L419fs	L419fs	L419fs	L419fs, D423fs^$^
*MAP2K3*	P40T^$^	P40T^$^	P40T^$^	P40T^$^	P40T^$^
*CDK11A/B*	R117delinsKER	R117delinsKER	R117delinsKER	R117delinsKER	R117delinsKER
*MAP3K4*	p.1186_1186del	p.1186_1186del	p.1186_1186del	p.1186_1186del	p.1186_1186del
*WNK1*	V724fs^$^	P1989A^$^	V724fs^$^, R1076Q^$^	V724fs^$^, R1076Q^$^	
*FPGT-TNNI3K*	P830T^$^	M931V^$^	–	M931V^$^	T751M^$^
*LIMK2*	Q684R		Q684R	Q684R	Q684R
*TTN*	T1968A^$^, R8985C^$^, P18296A^$^, R24838H^$^	E8785Q^$^	V5249F^$^, M11124T^$^	V5249F^$^, M11124T^$^	–
*WNK2*	V828M	V828M	–	–	A690P^$^, V828M, S1789fs^$^
*TGFBR2*	–	R522X	S320X	S320X	–
*TNK2*	–	R162W	–	–	R162W
Transcription factors					
*BCLAF1*	G64A^$^, T837N^$^	G64A^$^, R697H^$^, S716R^$^, T837N^$^	G64A^$^, G208V^$^, T837N^$^, R864C^$^	G64A^$^, T837N^$^	G64A^$^, R697H^$^, S716R^$^, T837N^$^, R864C^$^
*ZBTB33*	S188delinsSD	S188delinsSD	S188delinsSD	S188delinsSD	S188delinsSD
*MAML3*	Q498fs^$^, Q502fs^$^, p.768_768del	Q498fs^$^, Q502fs^$^, p.768_768del	Q498fs^$^, Q502fs^$^, Q505fs, p.768_768del	Q498fs^$^, Q502fs^$^, p.768_768del	Q505fs, p.768_768del
*FOXE1*	p.169_170del	p.169_170del	p.169_170del	p.169_170del	p.169_170del
*DCP1A*	Splicing^$^	Splicing^$^	Splicing^$^	Splicing^$^	Splicing^$^
*CREB3L1*	Splicing^$^	Splicing^$^	Splicing^$^	Splicing^$^	Splicing^$^
*TBP*	p.52_52del	p.52_52del	p.52_52del, p.56_56del^$^	p.52_52del, p.56_56del^$^	–
*PRDM2*	T501delinsTP	T501delinsTP	–	T501delinsTP	C133Y^$^, T501delinsTP
*ZNF83*	p.267_295del	p.267_295del	–	p.267_295del	–
*SMARCA2*	–	p.223_223del	p.223_223del	–	p.223_223del
*CASZ1*	P1738R^$^	G1732S^$^	–	R277W	–
*NCOA3*	–	–	p.1247_1249del	p.1247_1249del	p.1254_1254del
*MEF2A*	p.342_342del	–	p.342_343del	p.342_343del	–
*HOXD9*	P265delinsPQ	–	–	–	P265delinsPQ
*IRF5*	p.168_177del	p.168_177del	–	–	–
*ZNF169*	R382C	P72L	–	–	–
*PRDM10*	–	D180V^$^	–	–	L930V^$^
*NR1D2*	–	Q232H^$^	–	–	F96Y^$^
*FAM20C*	L317_D318delinsLDRX^$^	L317_D318delinsLDRX^$^	–	–	–
*EP300*	–	–	S507G	S507G	–
*STAT2*	–	–	F90S^$^	F90S^$^	–
Other important genes					
*CD207*	Splicing^$^	Splicing^$^	Splicing^$^	Splicing^$^	Splicing^$^
*SIRPA*	D95fs^$^	D95fs^$^	D95fs^$^	D95fs^$^	D95fs^$^
*LILRB3*	V341M^$^	–	V341M^$^	V341M^$^	V341M^$^
*PDE4DIP*	Q1665fs^$^	L764fs^$^	–	–	L764fs^$^
*CD33*	N237fs^$^	–	N237fs^$^	N237fs^$^	–
*SELPLG*	p.151_161del	–	p.151_161del	p.151_161del	–

*Note*: X stop codon, * validated by Sanger sequencing, ^Δ^ not validated by Sanger sequencing, other variations (without labels * or ^Δ^) have not been tested by Sanger sequencing, ^$^ variants that were not indexed in the COSMIC70 database

Notably, cell lines 149RCa and 149RM had similar gene variant calls, except few mutations, such as *BCL11A*:E539K, *CASZ1*:R277W in 149RM, and *FBXW7*:S25X, *NOTCH2*:S220R/P227H/T235S, *BCLAF*:G208V/R864C, and *NIN*:C1036F in 149RCa. Whether these unique variations are relevant to their biological differences (such as motility) requires further investigations. Additionally, we detected several deleterious or possibly damaging variants in PSCC cell lines, which were not indexed in the COSMIC database (Table 1). Detailed SNV/InDel information of important functional genes in cell lines and corresponding tumor tissues was shown in Additional Tables S5–S9.

### Identification of prioritized copy number variations (CNVs) in penile cancer cell lines

CNV analyses showed that five cell lines presented different amplification/deletion profiles, the whole-genomic CNVs are shown in Fig. 5c. Further analyses revealed that high- frequency gains of oncogenes *MYC* (5/5), *PLAG1*, *NCOA2*, *RUNX1T1*, *COX6C*, and *CHCHD7* (4/5), *HOXA13* (3/5), *EGFR*, *HOXA9*, *HOXA11*, *ETV1* and *PIK3CA* (2/5), and losses of tumor suppressors *FBXW7*, *TET2*, *XPC*, and FANCE (2/5) were frequently observed in these cell lines. Surprisingly, we also found that there were partial losses of certain oncogenes (FGFR1, PDGFRA, and so on) and amplifications of certain tumor suppressors (NBN, EXT1, and so on) in these cell lines. However, due to the complexity of molecular interaction in cancer, the roles that these changes play in penile carcinoma need a further investigation. CNV profiles of critical functional genes in these cell lines are shown as an integrative heatmap in Fig. 5d, and detailed CNVs information of important functional genes in each cell lines was shown in Additional Tables S10–S14.

### Aberrant pathways in penile cell lines

We combined SNVs, InDels, and CNVs to perform KEGG pathway analysis, and determined that genomic alterations in these cell lines were mainly involed in the MAPK, Jak-STAT, TGF-beta, Notch, and apoptosis signaling pathways. Main aberrant pathways and involved gene variations were showed in Table 2.

**Table 2:** Critical aberrant pathways in penile cell lines

Cell line	Pathway	Involved gene variations
Penl1	Jak-STAT signaling pathway	Copy number gain (MYC, IL7R, LIFR, JAK2, IFNA4, IFNA2, IFNA5, IFNA7, IFNA8, IFNA14, IFNA16, IFNA21, IFNW1); Copy number loss (IL2RA, IL5RA, IL9R, IL15, IL10RB, IL21, PIAS2)
	TGF-beta signaling pathway	Copy number gain (MYC, BMP2, GDF5, RBL1, ID1); Copy number loss (TGFBR2, BMPR1B, ACVR2B, SMAD4, SMAD2, SMAD1, SMAD7, PITX2); TGFB3:NM_003239:exon1:c.202C>T:p.Q68X
	MAPK signaling pathway	Copy number gain (MYC, FGF10); Copy number loss (TGFBR2, EGF, PDGFRA, NFKB1, IKBKB, RAF1, MAPK10, FGFR1, FGF2, FGF5, FGF17, MAP3K8, MAPKAPK3); TGFB3:NM_003239:exon1:c.202C>T:p.Q68X; IL1R2:NM_004633:exon8:c.989A>G:p.N330S; MAP2K3:NM_145109:exon3:c.118C>A:p.P40T; MAP3K3:NM_203351:exon11:c.934G>A:p.V312M
	Notch signaling pathway	Copy number gain (JAG1); Copy number loss (MAML3); NOTCH1:NM_017617:exon8:c.1280G>A:p.G427D; NOTCH2:NM_024408:exon2:c.137A>G:p.N46SDTX2; NCOR2:NM_006312:exon46:c.6943G>A:p.A2315T; MAML3:NM_018717:exon2:c.1494_1504del:p.Q498fs, exon2:c.1506delG:p.Q502fs, exon4:c.2302_2304del:p.768_768del
	Apoptosis	Copy number gain (BIRC3); Copy number loss (NFKB1, IKBKB, TNFRSF10B, TNFRSF10D, TNFRSF10A, BCL2, IRAK4, IRAK2); TP53:NM_001126118:exon7:c.731G>C:p.R244P, exon3:c.98C>G:p.P33R
Penl2	MAPK signaling pathway	Copy number gain (MYC, FGF10, FGF12, FGF13, FGF14, MECOM, STK4, PRKCG, PAK2, MOS, RPS6KA6, RPS6KA3, ELK1, STK3, MAP3K13); Copy number loss (MAPK3, MAP2K2, FOS, FGFR1, FGF9, FGF22, FGF18, FGF17, PRKACA, NFATC4, MAPK12, MAPK11, MAPK13, RPS6KA2, MAPKAPK3, MKNK2, SRF, MAP3K14, CD14, FGFR4, ATF4, TAOK2); MAP2K3:NM_145109:exon3:c.118C>A:p.P40T; MAP3K4:NM_001301072:exon17:c.3554_3556del:p.1186_1186del
	Jak-STAT signaling pathway	Copy number gain (MYC, PIK3CA, STAT3, IL5, IFNA2, IFNA21, IL7, IFNW1, IL13RA1, IL13RA2); Copy number loss (PDGFRB, TGFB1, TGFB3, AKT2, CSF2, IL3, IL13, IL4R, IL9R, CSF2RB, IL2RB, GH1, GH2, LIF, OSM, STAT5A, JAK3, CBLC, IRF9); CSF2:NM_000758:exon4:c.356C>A:p.T119N; TGFBR2:NM_001024847:exon7:c.1564C>T:p.R522X; IL12RB1:splicing
	TGF-beta signaling pathway	Copy number gain (MYC, E2F5, RBL1, GDF6); Copy number loss (MAPK3, TGFB1, TGFB3, BMP2, AMH, E2F4, ID3, SMAD9); TGFBR2:NM_001024847:exon7:c.1564C>T:p.R522X
	Notch signaling pathway	Copy number gain (HES1); Copy number loss (KAT2A, NUMBL, NCOR2); NOTCH1:NM_017617:exon8:c.1400G>T:p.C467F; NOTCH2:NM_001200001:exon1:c.17_18del:p.P6fs,NOTCH2:NM_024408:exon1:c.17_18del:p.P6fs; DTX2:NM_020892:exon8:c.1151G>A:p.G384E; MAML3:NM_018717:exon2:c.1494_1504del:p.Q498fs, exon2:c.1506delG:p.Q502fs, exon4:c.2302_2304del:p.768_768del; NUMBL:NM_001289979:exon9:c.1182_1187del:p.394_396del; NCOR2:NM_006312:exon16:c.1531_1532insAGC:p.P511delinsQP, exon39:c.5517_5518insAGCAGCGGC:p.G1840delinsSSGG
	Apoptosis	Copy number gain (PIK3CA, TNFSF10); Copy number loss (AKT2, IL3, CSF2RB, PRKACA,MAP3K14, TNFRSF10A, NFKBIA, RIPK1); TP53:NM_001126118:exon7:c.701G>A:p.R234H; ATM:NM_000051:exon55:c.8071C>T:p.R2691C
149RCa	MAPK signaling pathway	Copy number gain (MYC, EGFR, PDGFA, FGF13, FGF16, MOS, RPS6KA6, RPS6KA3, STK3, PRKX); Copy number loss (TNF, MAPK10); HRAS:NM_001130442:exon2:c.38G>T:p.G13V; FGFR4:NM_022963:exon4:c.535A>G:p.T179A; MAP2K3:NM_145109:exon3:c.118C>A:p.P40T; MAP3K4:NM_001301072:exon17:c.3554_3556del:p.1186_1186del; EGF:NM_001178130:exon1:c.46A>C:p.S16R; FGF6:NM_020996:exon1:c.251G>A:p.R84Q; FGF18:NM_003862:exon5:c.472C>T:p.R158W; TGFBR2:NM_001024847:exon5:c.959C>G:p.S320X
	Jak-STAT signaling pathway	Copy number gain (MYC, CSF2RA, IL3RA, IL6, CRLF2, IL7, IL13RA1, IL13RA2, IL5, IFNW1, IFNA2); STAT2:NM_005419:exon3:c.269T>C:p.F90S; EP300:NM_001429:exon6:c.1519A>G:p.S507G; LIFR:NM_001127671:exon2:c.116T>A:p.M39K; CSF3R:NM_000760:exon17:c.2197C>A:p.P733T
	TGF-beta signaling pathway	Copy number gain (MYC, E2F5, INHBA); Copy number loss (TNF, BMP4, PITX2); EP300:NM_001429:exon6:c.1519A>G:p.S507G; TGFBR2:NM_001024847:exon5:c.959C>G:p.S320X; ACVRL1:NM_001077401:exon5:c.706G>A:p.E236K; LTBP1:NM_000627:exon15:c.2048C>A:p.P683Q
	Notch signaling pathway	NOTCH2:NM_001200001:exon2:c.137A>G:p.N46S, exon4:c.660C>A:p.S220R, exon4:c.680C>A:p.P227H, exon4:c.703A>T:p.T235S; EP300:NM_001429:exon6:c.1519A>G:p.S507G; MAML3:NM_018717:exon2:c.1494_1504del:p.Q498fs, exon2:c.1506delG:p.Q502fs, exon3:c.1513_1514del:p.Q505fs, exon4:c.2302_2304del:p.768_768del; Copy number loss (DTX2)
	Apoptosis	Copy number gain (IL3RA, BIRC3, PRKX, IRAK1); Copy number loss (TNF); TP53:NM_001126118:exon3:c.98C>G:p.P33R, exon3:c.211C>T:p.R71C, exon6:c.626G>A:p.R209Q; RIPK1:NM_003804:exon9:c.1335T>A:p.H445Q
149RM	MAPK signaling pathway	Copy number gain (MYC, EGFR, PDGFA, AKT3, MOS, STK3, TGFB2, FGF19, ELK4, MAP3K13); Copy number loss (MAPK10, AKT1, IKBKB, NFKB1, PDGFRA, TGFB3, FGFR1, FGF2, FGF5, FGF20, FGF17, FOS, RPS6KA5, RPS6KA1, MAPKAPK3, MAP3K6); HRAS:NM_001130442:exon2:c.38G>T:p.G13V; MAP2K3:NM_145109:exon3:c.118C>A:p.P40T; MAP2K6:NM_002758:exon4:c.139G>C:p.E47Q; MAP3K4:NM_001301072:exon17:c.3554_3556del:p.1186_1186del; EGF:NM_001178130:exon1:c.46A>C:p.S16R; FGF6:NM_020996:exon1:c.251G>A:p.R84Q; FGF18:NM_003862:exon5:c.472C>T:p.R158W; GFBR2:NM_001024847:exon5:c.959C>G:p.S320X
	Jak-STAT signaling pathway	Copy number gain (MYC, PIK3CA, IL6, IL7, IL5, AKT3); Copy number loss (AKT1, IL2, IL5RA, IL15, IL10RB, PIAS2); STAT2:NM_005419:exon3:c.269T>C:p.F90S; EP300:NM_001429:exon6:c.1519A>G:p.S507G; LIFR:NM_001127671:exon2:c.116T>A:p.M39K
	TGF-beta signaling pathway	Copy number gain (MYC, E2F5, INHBA, TGFB2, LEFTY1, GDF6, LEFTY2); Copy number loss (TGFB3, ROCK1, BMPR1B, PITX2, ACVR2B, SMAD4, SMAD2, SMAD1, SMAD7, ID3); EP300:NM_001429:exon6:c.1519A>G:p.S507G; TGFBR2:NM_001024847:exon5:c.959C>G:p.S320X; ACVRL1:NM_001077401:exon5:c.706G>A:p.E236K; LTBP1:NM_000627:exon15:c.2048C>A:p.P683Q
	Notch signaling pathway	NOTCH2:NM_001200001:exon2:c.137A>G:p.N46S; EP300:NM_001429:exon6:c.1519A>G:p.S507G; MAML3:NM_018717:exon2:c.1494_1504del:p.Q498fs, exon2:c.1506delG:p.Q502fs, exon4:c.2302_2304del:p.768_768del; Copy number loss (HDAC1, SNW1, RBPJ); Copy number gain (HES1)
	Apoptosis	Copy number gain (BIRC3, AKT3, PIK3CA, TNFSF10); Copy number loss (AKT1, IKBKB, NFKB1, TNFRSF10B, TNFRSF10D, TNFRSF10C, TNFRSF10A, BCL2); TP53:NM_001126118:exon3:c.98C>G:p.P33R, exon3:c.211C>T:p.R71C, exon6:c.626G>A:p.R209Q; RIPK1:NM_003804:exon9:c.1335T>A:p.H445Q
LM156	Jak-STAT signaling pathway	Copy number gain (MYC, IL7); Copy number loss (IFNA1, IFNA2, IFNA4, IFNA5, IFNA6, IFNA7, IFNA8, IFNA10, IFNA13, IFNA14, IFNA16, IFNA17, IFNA21, IFNB1, IFNGR1, IFNW1, IFNK); IL5RA:NM_175725:exon6:c.284C>G:p.S95X; PRL:NM_000948:exon4:c.391C>T:p.R131C; PIK3CA:NM_006218:exon12:c.1834C>T:p.R612X; JAK3:NM_000215:exon16:c.2062A>T:p.I688F
	MAPK signaling pathway	Copy number gain (MYC, MOS, STK3, STK4); Copy number loss (PRKCG, IKBKB, MAP3K7, FGFR1, FGF20, FGF17, MAP3K5, RPS6KA2, MAP3K4, TAOK2); HRAS:NM_176795:exon2:c.37G>C:p.G13R; NFATC2:NM_012340:exon4:c.1337A>G:p.H446R; MAP2K3:NM_145109:exon3:c.118C>A:p.P40T; MAP3K4:NM_005922:exon17:c.3566_3568del:p.1189_1190del; MAPKAPK5:NM_139078:exon14:c.1331G>A:p.R444H
	Notch signaling pathway	NOTCH1:NM_017617:exon22:c.3598G>A:p.D1200N; NOTCH2:NM_024408:exon2:c.137A>G:p.N46S; KAT2A:NM_021078:exon8:c.1181_1186del:p.394_396del; MAML3:NM_018717:exon3:c.1513_1514del:p.Q505fs, exon4:c.2302_2304del:p.768_768del; Copy number gain (DTX2); Copy number loss (HDAC2)
	TGF-beta signaling pathway	Copy number gain (MYC, E2F5, BMP2, GDF6, RBL1); Copy number loss (BMP6, ID4); BMP6:NM_001718:exon2:c.695A>C:p.Q232P
	Apoptosis	TP53:NM_001126118:exon3:c.98C>G:p.P33R; PIK3CA:NM_006218:exon12:c.1834C>T:p.R612X; Copy number loss (IKBKB, TNFRSF10B, TNFRSF10D, TNFRSF10C, TNFRSF10A, BCL2)

### Genomic structural variations (SVs) in penile cancer cell lines

The distribution and number of SVs are exhibited in Fig. 5e and Additional Table S15. LM156 cells possessed the largest number of SVs (352), while the other four cell lines had similar SV numbers (from 144 to 200). The inter-chromosomal translocation (CTX) and deletion (DEL) were the most frequent SV types. However, no fusion genes were found in the five cell lines.

## Discussion

Cell line models are essential tools to study the molecular mechanisms underlying the tumor progression, metastasis, relapses, and treatment resistance^17–20^. Various tumors, even same tumor, have been confirmed as being driven from different molecular mechanisms^17–19^, so it is necessary to obtain cell line models with identified genetic characteristics for the PSCC study. Although a few PSCC cell lines were developed several years ago^10–14^, little molecular mechanisms underlying PSCC initiation and progression have been clarified using these cell line models.

We have successfully established a panel of PSCC cell lines including Penl1^15^ and four additional PSCC cell lines: Penl2, 149RCa, 149RM, and LM156, well characterized them in genomic alterations by WGS assay. All cell lines consist of pure, immortalized, and epithelial-like cells without HPV infection and harbor some common or specific biological characteristics and genomic variations. These cell lines present different growth, migratory, invasive, and tumorigenic abilities. Among them, 149RM has the highest mobility but the lower invasion ability, as well as the shortest doubling time, which may contribute to the rapid wound healing. Three cell lines from metastatic LNs, especially Penl2, has stronger invasion potential, thus these cell lines would be better cell models for the investigation of mechanisms underlying LN metastasis in penile cancer. Although 149RM from a nearby scrotal invasion lesion was expected to be more aggressive, it has a lower invasion rate. We supposed that this scrotal lesion was originated by direct contact of primary tumor, but not by invasion of tumor cells. Intriguingly, 149RM had a similar doubling time as 149RCa, whereas the 149RM xenograft tumors were smaller than 149RCa. We presumed that the microenvironment in vivo play more important role in xenografts’ growth, the underlying mechanisms need to be further explored.

The WGS data of cell lines revealed that C:G>T:A and T:A>C:G transitions were the most frequent substitution modes in PSCC cells, which were consistent with previous genomic profiling analysis in penile cancer^21^. Interestingly, this mutation pattern differed from that in other solid tumor^22^. This PSCC-specific mutation signature and the etiology of penile cancer need further investigation. Mutation or loss of *CDKN2A* gene, *TP53* mutation, RAS mutation, amplifications of *MYC* and *EGFR* genes have been reported in many human malignancies^23–26^, including penile carcinoma^9,27^, and the clinical data imply that the loss-of-function mutations of tumor suppressor genes and activating variations of oncogenes play a critical role in the carcinogenesis and invasion of PSCC^9,28^. We found that all five cell lines harbor *CDKN2A* deletion/mutation, *MYC* amplification, and *TP53* mutations, which strongly support the above findings and hypothesis. These findings are also in accordance with Ali et al.’s comprehensive genomic profiling analysis, which involved 3769 exons of 236 cancer-related genes plus 47 introns from 19 genes frequently rearranged in cancer^7^.

PSCC is a male-specific malignancy, which may be similar to prostate cancer that correlates with androgen stimulation and AR expression^29^. Marchi et al. found that AR was one of top ten driver candidates in penile cancer by a multidimensional integrative analysis^30^. However, compared to normal glans tissues, AR expression in penile cancer tissue was greatly downregulated by increased microRNAs^30^ or promoter hypermethylation^31^, which is in accord with our result that the five cell lines established in our laboratory expressed very low levels of AR. It seems that androgen stimulation plays a negative role in penile cancer. Additionally, we found that penile cancer cells did not response to androgen stimulation in vitro. So we are unable to identify whether AR plays a driver candidate role or a passenger role, which needs to be validated in future functional studies.

Our further investigations revealed that all five PSCC cell lines were sensitive to cisplatin, a DNA-damaging anti-cancer drug used in the clinic^32^. Notably, *ERCC5* mutations in all of five PSCC cell line, the mutations of *FANCD2* in four of five PSCC cell lines, *ATM* and *MSH6* mutations and *BRCA2* loss in Penl2, and loss or stop-gain mutations of *FBXW7* in Penl1, 149RM, and 149RCA were found, and these genes are all important DNA damage repair-related genes^33–35^, which may well account for the cisplatin sensitivity of these cell lines. Therefore, the status of DNA damage repair-related genes may be a promising marker to identify PSCC patients who can benefit from DNA-damaging chemotherapy.

Previous studies have shown that *EGFR* is frequently overexpressed in PSCC, and anti-EGFR therapy may be a potential therapeutic option for PSCC patients^36^. We detected *EGFR* amplification in two cell lines, and *EGFR* overexpression in most of these cell lines, but they were all resistant to EGFR inhibitors. Activating *HRAS* and *PI3KCA* mutations were found to correlate with resistance to anti-EGFR therapy^37,38^, so we presume that *HRAS* G13V/G13R mutations in 149RCa, 149RM, and LM156 cells and *PI3KCA* amplification in Penl2 and 149RM cells may relate with their resistance to anti-EGFR therapy, which need to be verify in further investigations. Thus, these PSCC cell lines may be helpful models for further investigation of the mechanisms underlying anti-EGFR resistance and strategies overcoming this resistance in PSCC.

In summary, we established a panel of cell lines derived from PSCC patients with different clinicopathological characteristics, and these cell lines display diverse biological characteristics. WGS analysis revealed that these cell lines harbor a genomic alteration spectrum similar to that in PSCC patients reported by previous studies, and they display their specific gene alterations and amplification/loss spectrums, suggesting that this panel of PSCC cell lines might be suitable model systems for PSCC research. The comprehensive biological and genetic information should aid researchers in the selection of representative penile cancer cell lines for investigating molecular mechanisms and screening potential therapeutics.

## Materials and methods

### Cell line establishment and morphological examination

Fresh tumor specimens were obtained from patients in the Department of Urology, Sun Yat-Sen University Cancer Center (SYSUCC). Four new cell lines Penl2, 149RCa, 149RM, and LM156 were established using the same procedure as Penl1, developed previously in our laboratory^15^. To remove cancer-associated fibroblasts, cells were briefly digested with 0.25% trypsin/ethylenediaminetetraacetic acid (Invitrogen) for 4–5 times as previously reported^15,39^. Cells were cultured in DMEM supplemented with 10% FBS (Gibco, Life Technologies) at 37 °C with humidified air and 5% CO_2_, subcultured at a ratio of 1:3 to 1:4 and cryopreserved in complete growth medium supplemented with 10% (v/v) dimethyl sulfoxide at 1 × 10^6^ cells/mL in liquid nitrogen. All PSCC cell lines were continuously passaged every 3 days for over 50 passages, without any exogenous transfection or stimulation. Cell lines at passage 15–25 were used for further analyses. Cell morphology of cell lines was examined and photographed under a phase-contrast microscope.

### Flow cytometry assay

Cells were harvested by trypsinization, fixed with BD Cytofix/Cytoperm solution (BD Bioscience) and stained with Alexa Fluor^®^ 488-conjugated anti-Pan-CK antibody or corresponding mouse (DA1E) mAb IgG XP^®^ isotype control (1:50, Cell Signaling Technology) for 30 min at room temperature, followed by a flow cytometry analysis. The results were analyzed with Kaluza software (Beckman Coulter Inc.). The percentage of cells with Pan-CK positive represented the purity of cell lines^15^. The experiments were independently performed for three times, and representative images were shown.

### WB assay

WB assay was performed as previously described^40,41^. Briefly, protein samples from cell lysates were separated by sodium dodecyl sulfate-polyacrylamide gel electrophoresis and transferred to polyvinylidene fluoride membranes. After blocking for non-specific binding, the blots were inoculated with primary antibodies against EGFR (1:2000, Cell Signaling Technology), Vimentin, Pan-CK, AR and glyceraldehyde phosphate dehydrogenase (GAPDH) (1:1000, Santa Cruz Biotechnology), followed by reaction with corresponding HRP-conjugated secondary antibodies. Bands were visualized via Chemidoc Touch (Bio-Rad).

### Polymerase chain reaction (PCR)-based assays

Genome DNA was extracted from cell lines and corresponding tumor tissues, and subjected to analyze the infection of HPV and mycoplasma and the STR profiles. HPV infection was analyzed in MyGenostics Inc. (Beijing, China) by quantitative real-time PCR assays with specific primers and probes (Additional Table S16) as described previously, which can detect both free and integrated HPV DNA^42^. Mycoplasma contamination using the PCR Mycoplasma Test Kit (HuaAn Biotech, Hangzhou, China) according to the manufacturer’s instruction^15,39^.

The STR profile analysis was performed by PCR amplification^43^ using Goldeneye 20A kit (Peoplespot Inc., China) and then processing with the ABI3730xl Genetic Analyzer. The STR profile of each cell line was compared with those from the ATCC, DSMZ, or JRCB databases for reference matching.

### Cell proliferation and viability assays

For cell proliferation assay, 5 × 10^3^–1 × 10^4^ cells were seeded in 24-well plates in triplicate. Cell were harvested and counted after typan blue staining^44^ using a Beckman Z2 Coulter^®^ Particle Count and Size Analyzer (Beckman Coulter). Cell growth curve was analyzed in an exponential growth phase using Graphpad 5.0 software to obtain cell doubling time.

Cell viability assay was performed as described previously. Briefly, 5000 cells/well were seeded in 96-well plates for 24 h, and then exposed to androgen (Sigma, USA), cisplatin (Luoxin Biotechnology, China), cetuximab, erlotinib, or afatinib (Selleck, China). Cell viability was measured by cell counting kit (CCK)-8 (Dojindo, Japan) assay after 48 h. IC_50_ values were calculated using Graphpad Prism 5.

### In vitro migration and invasion assay

The migration ability of cell lines was assessed using a monolayer wound healing assay^45^. Briefly, cells were seeded in 6-well plates and grown to confluence overnight. Cells were slightly scratched with a 200 μL-tip and then washed twice with phosphate buffered saline. Fresh serum-free medium were then added and wound healing was monitored after 12 h of incubation.

Cell invasion was tested using a Transwell assay^46,47^. Briefly, 2 × 10^5^ cells in 200 μL serum-free or complete medium were seeded into the Boyden chamber with Matrigel (8-μm pore; BD Falcon, USA). The chambers were put in 24-well plates with medium with 10% FBS to incubate for 24 h. The cells attached to the undersurface of filter membrane were fixed and stained in 0.1% crystal violet. The invaded cells were counted in five random fields (100×) under a microscope.

### Tumorigenicity in nude mice

To confirm the tumorigenicity of these cell lines, 5 × 10^6^ cells (passage 20–25) suspended in 100 μL 20% Matrigel (BD Biosciences, USA) were injected subcutaneously into the right flanks of 6-week-old female and male nude mice (10 mice per cell line). After 3 weeks, mice were sacrificed and tumors were removed for H&E staining and pathology examination.

### WGS and whole-exome sequencing (WES)

WGS analysis for PSCC cell lines (90 Gb PF data for each cell line), WES analysis was performed for cell line-corresponding cancer tissues (Agilent’s SureSelect Human All Exon v5 fors exome capture, 10G PF data for each tissue) were performed by Wuxi Apptec (Shanghai, China) using the Illumina HiSeq X sequencing system. The raw data of WGS/WES in this study were deposited at the NCBI (SRA accession number: SRP117294). High-quality reads were aligned against the NCBI human reference genome (hg19) using Burrows-Wheeler Aligner (v0.5.9) with default parameters, followed by passing a hard filter (the detailed description was showed in Additional Methods).

CNVs were calculated based on the read depth of WGS, SVs were analyzed and annotated by the chromosomal locations, while SNVs and InDels were identified by a customized bioinformatics data analysis pipeline. Prioritized functional SNVs and InDels were identified as meet one of the following criteria: (1) Splicing site, frameshift insertion/deletion, stop-gain or stop-loss mutations that are excluded in SNP142common database; (2) Non-synonymous mutations that are excluded in SNP142common database, which functional impacts were “deleterious (D)” predicted by SIFT or “probably damaging/possibly damaging (P/D)” predicted by PolyPhen2; (3) Non-frameshift insertion/deletion mutations that are included in COSMIC (Catalog of Somatic Mutations in Cancer) 70 database and excluded in SNP142common database; (4) SNVs and InDels included in SNP142common database and COSMIC70 database, which functional impacts were “D” predicted by SIFT or “P/D” predicted by PolyPhen2.

### Statistical analysis

All of in vitro experiments were performed in triplicate and repeated three times and animal experiments were repeated twice. Heatmap Illustrator (HemI 1.0)^48^, SPSS 20.0, and GraphPad 5.0 software programs were used for statistical analyses in this study. One-way ANOVA was utilized to compare the differences among the five cell lines while Student’s *t*-test was used to compared the difference between two groups. *p* < 0.05 was considered as significantly different.

## Electronic supplementary material

Additional Fig S1-S3

Additional Table S1

Additional Table S2

Additional Table S3

Additional Table S4

Additional Table S5

Additional Table S6

Additional Table S7

Additional Table S8

Additional Table S9

Additional Table S10

Additional Table S11

Additional Table S12

Additional Table S13

Additional Table S14

Additional Table S15

Additional Table S16

Additional Methods
