# Supplementary material for: Molecular characterization and integrative genomic analysis of a panel of newly established penile cancer cell lines
Source: Cell Death Dis. 2018 Jun 7;9(6):684. doi: 10.1038/s41419-018-0736-1 (PMC5992159; doi:10.1038/s41419-018-0736-1)
Supplement: Supplementary file 1 — Additional Fig S1-S3 [file 41419_2018_736_MOESM1_ESM.docx]

**Additional Figures**

**
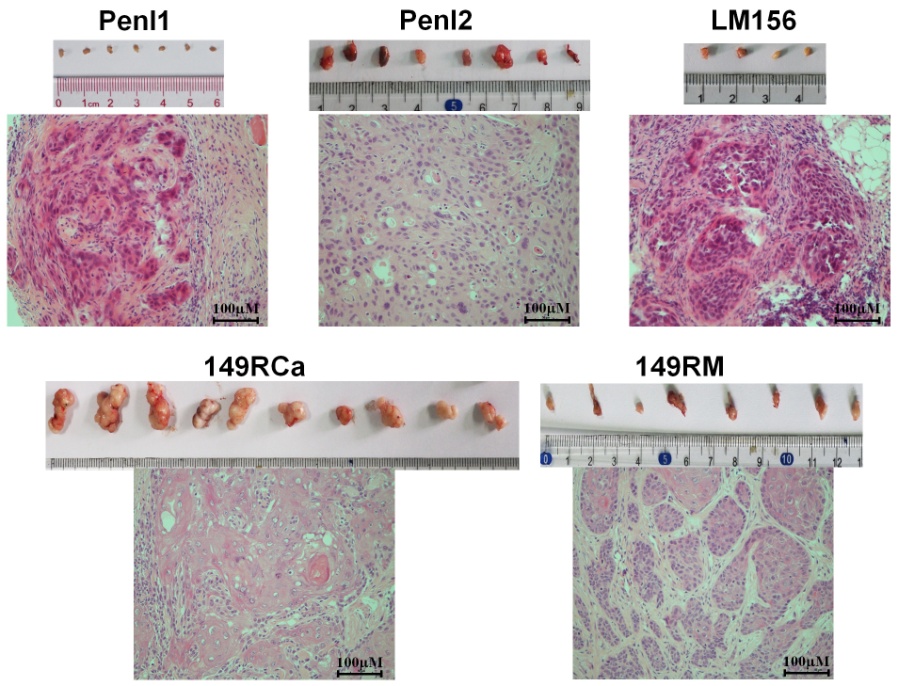
**

**Additional Figure S1:** Xenograft tumors harvested 3 weeks after tumor cells inoculation (in female nude mice) and the corresponding hematoxylin and eosin staining pictures of xenograft tumors, confirming the histology of SCC.

**
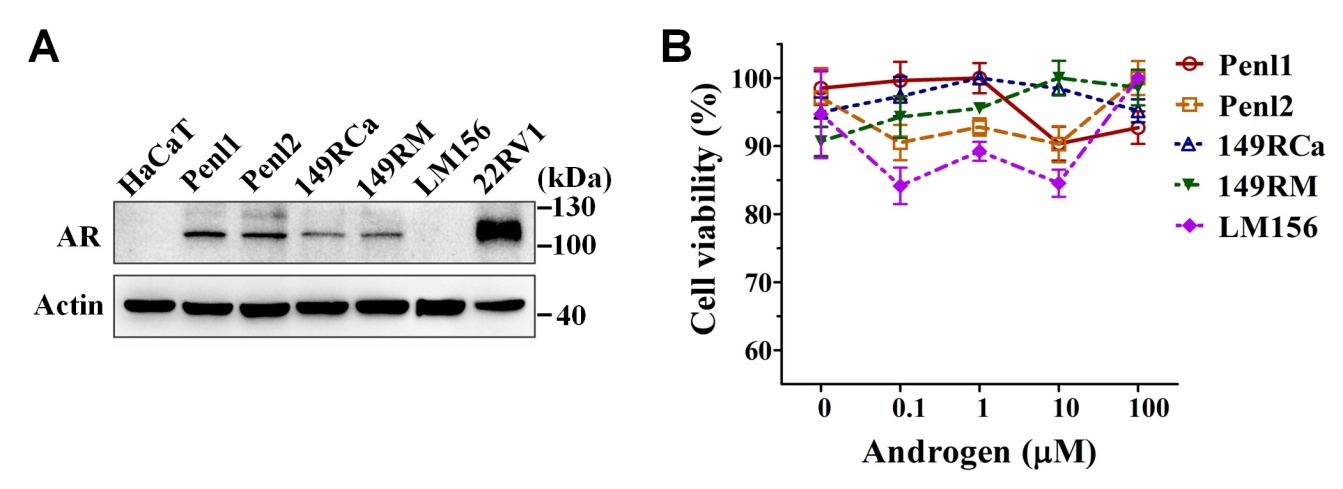
**

**Additional Figure S2: The response of penile cancer cell lines to Androgen.** Cells were treated with Androgen for 48 hours. A CCK-8 assay was used to assess the relative cell viability. (**A**) WB assays for androgen receptor (*AR*) expression (actin was used as the loading control). (**B**) Representative cell viability curve after androgen treatment.

**
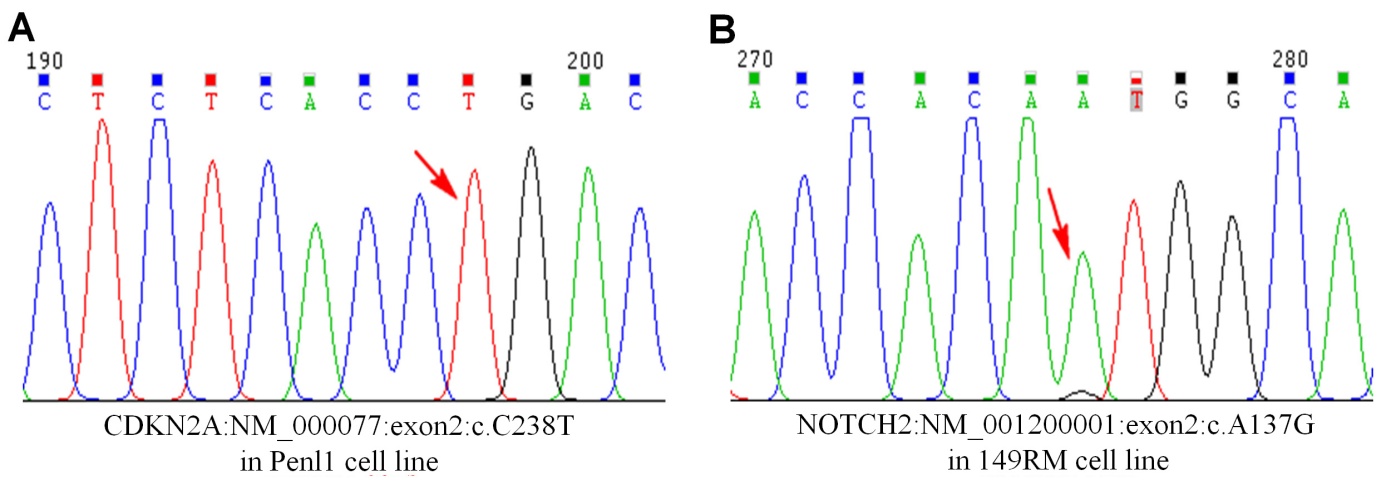
**

**Additional Figure S3: The WGS results were validated by Sanger sequencing.** Representative chromatograms of Sanger sequencing. (**A)** *CDKN2A*:NM_000077 exon2:c.C238T: pR80X in Penl1 cell line, showing a homozygous C>T mutation that is in accordance with WGS data. (**B**) *NOTCH2*: NM_001200001 exon2:c.A137G in 149RM cell line, showing only a very low G peak (mutation) under a high A peak (reference)). Because the G peak is too low, so we think it is not accordance with a heterozygotic A>G mutations in WGS. Red arrow indicated the predicted mutation site in WGS data.
