# Supplementary material for: Molecular characterization and integrative genomic analysis of a panel of newly established penile cancer cell lines
Source: Cell Death Dis. 2018 Jun 7;9(6):684. doi: 10.1038/s41419-018-0736-1 (PMC5992159; doi:10.1038/s41419-018-0736-1)
Supplement: Supplementary file 18 — Additional Methods [file 41419_2018_736_MOESM18_ESM.docx]

**Additional Methods:**

SNPs matching any of these conditions will be considered bad and filtered out, marked FILTER in the output VCF file. The program will specify which parameter was chiefly responsible for the exclusion of the SNP using the culprit annotation. SNPs that do not match any of these conditions will be considered good and marked PASS in the output VCF file.

(1) QualByDepth (QD) 2.0: This is the variant confidence (from the QUAL field) divided by the unfiltered depth of non-reference samples.

(2) FisherStrand (FS) 200: Phred-scaled p-value using Fisher’s Exact Test to detect strand bias (the variation being seen on only the forward or only the reverse strand) in the reads. More bias is indicative of false positive calls.

(3) RMSMappingQuality (MQ) 40.0: This is the Root Mean Square of the mapping quality of the reads across all samples.

(4) ReadPosRankSumTest (ReadPosRankSum) -20: This is the u-based z-approximation from the Mann-Whitney Rank Sum Test for the distance from the end of the read for reads with the alternate allele. If the alternate allele is only seen near the ends of reads, this is indicative of error. Note that the read position rank sum test cannot be calculated for sites without a mixture of reads showing both the reference and alternate alleles, i.e. this will only be applied to heterozygous calls.

In/Dels matching any of these conditions will be considered bad and filtered out, i.e. marked FILTER in the output VCF file. The program will specify which parameter was chiefly responsible for the exclusion of the indel using the culprit annotation. Indels that do not match any of these conditions will be considered good and marked PASS in the output VCF file.

(1) QualByDepth (QD) 2.0:This is the variant confidence (from the QUAL field) divided by the unfiltered depth of non-reference samples.

(2) FisherStrand (FS) 200.0: Phred-scaled p-value using Fisher’s Exact Test to detect strand bias (the variation being seen on only the forward or only the reverse strand) in the reads. More bias is indicative of false positive calls.

(3) ReadPosRankSumTest (ReadPosRankSum) -20.0: This is the u-based z-approximation from the Mann-Whitney Rank Sum Test for the distance from the end of the read for reads with the alternate allele. If the alternate allele is only seen near the ends of reads, this is indicative of error. Note that the read position rank sum test can not be calculated for sites without a mixture of reads showing both the reference and alternate alleles, i.e. this will only be applied to heterozygous calls.
